# Alkali-Activated Slag as Sustainable Binder for Pervious Concrete and Structural Plaster: A Feasibility Study

**DOI:** 10.3390/ma17164084

**Published:** 2024-08-17

**Authors:** Denny Coffetti, Simone Rapelli, Luigi Coppola

**Affiliations:** 1 Department of Engineering and Applied Sciences, University of Bergamo, Viale Marconi, 5, 24044 Dalmine, Italy; simone.rapelli@unibg.it (S.R.); luigi.coppola@unibg.it (L.C.); 2 CSGI Consortium, Research Center of Bergamo, Via della Lastruccia 3, 50019 Sesto Fiorentino, Italy; 3 INSTM Consortium, Research Unit of Bergamo, Via G. Giusti, 9, 50121 Firenze, Italy

**Keywords:** alkali-activated materials, alternative binders, pervious concrete, plaster, sustainability

## Abstract

In the realm of sustainable construction materials, the quest for low-environmental-impact binders has gained momentum. Addressing the global demand for concrete, several alternatives have been proposed to mitigate the carbon footprint associated with traditional Portland cement production. Despite technological advancements, property inconsistencies and cost considerations, the wholesale replacement of Portland cement remains a challenge. This study investigates the feasibility of using alkali-activated slag (AAS)-based binders for two specific applications: structural plaster and pervious concrete. The research aims to develop an M10-grade AAS plaster with a 28-day compressive strength of at least 10 MPa for the retrofitting of masonry buildings. The plaster achieved suitable levels of workability and applicability by trowel as well as a 28-day compressive strength of 10.8 MPa, and the level shrinkage was reduced by up to 45% through the inclusion of shrinkage-reducing admixtures. Additionally, this study explores the use of tunnel muck as a recycled aggregate in AAS pervious concrete, achieving a compressive strength up to 20 MPa and a permeability rate from 500 to 3000 mm/min. The relationship between aggregate size and the physical and mechanical properties of no-fines concretes usually used for cement-based pervious concrete was also confirmed. Furthermore, the environmental impacts of these materials, including their global warming potential (GWP) and gross energy requirement (GER), are compared to those of conventional mortars and concretes. The findings highlight that AAS materials reduce the GWP from 50 to 75% and the GER by about 10–30% compared to their traditional counterparts.

## 1. Introduction

Concrete is by far the most widely used man-made material in the building industry, and it is probably the key player in a world of growing urbanization. Thanks to its outstanding properties and ability to create complex shape elements, this material can be counted among the critical components in constructions as it is the bedrock of buildings (from single-floor houses to high-rise buildings), civil infrastructures (bridges, viaducts and tunnels), water and wastewater systems and power plants. Every year, more than 4.2 gigatonnes (Gt) of cement (about 2.8 Gt of ordinary Portland cement clinker and 1.4 Gt of fillers and supplementary cementitious materials, such as ground granulated blast furnace slag, fly ash and natural pozzolan), one of concrete’s main constituents, is produced, emitting about 38% of the about 10 Gt of carbon dioxide released globally by construction activities [1], approximately 450 kg per capita. In addition to CO_2_ emissions, dust, particulate matter, heavy metals and other greenhouse gases are also associated with OPC production. Other well-known environmental issues are related to the consumption of natural non-renewable resources and their local scarcity, energy use and water demand [2,3,4]. However, despite the strong environmental impact ascribable to the Portland cement and concrete industry, no materials seem to be able to replace reinforced concrete at a low economic cost. Taking into account the huge volumes involved, as well as matching its simplicity of use and outstanding properties, the replacement of Portland-based concrete appears unlikely in the next fifty years. As a consequence, it is essential to strongly promote the mitigation of environmental impacts from the concrete industry.

Various strategies have been proposed to mitigate the environmental footprint of ordinary Portland cement (OPC), which is the primary contributor to CO_2_ emissions in the concrete sector. This challenge is formidable because even with the use of ultra-efficient modern dry-process rotary kilns equipped with pre-calciner technology, the calcination of limestone accounts for approximately 65% of total carbon dioxide emissions (around 520 kg CO_2_ per ton of clinker), out of an average of 870 kg CO_2_ per ton of clinker. Therefore, the cement production industry is considered hard to abate, as decarbonizing the energy supply does not reduce the emissions derived from the calcination process.

Many studies have proposed to use alternative binders (i.e., calcium sulphoaluminate cements and alkali-activated materials) with a reduced environmental impact in comparison to that of conventional cements [5,6]. Among these, alkali-activated slag-based materials are very promising because, if properly designed, they exhibit a significantly higher strength than conventional Portland-based concretes and mortars, along with improvements in properties such as tensile strength [7]. Furthermore, by appropriately combining alkaline activators, it is possible to create composites with tailored mechanical properties, ranging from the typical strengths of plaster to those of concretes for structural reinforcement and high-strength mixtures [8]. However, the high shrinkage of AAS, if not adequately controlled with shrinkage-reducing admixtures (SRAs) and expansive agents, can compromise the durability, functionality, and aesthetics of products made with these innovative binders [9]. Additionally, the service life of structures manufactured with AAS exposed to different environments remains an open issue. Numerous tests have been conducted, but the results are often controversial, particularly when carbon steel is used as reinforcement [10,11,12]. The variability in test outcomes can be attributed to differences in experimental conditions, slag composition, and the nature of the activating solutions used. This inconsistency poses a challenge for establishing standardized guidelines and gaining broader acceptance within the construction industry.

Finally, to date, several issues limit the widespread use of these novel binders. The availability and cost of raw materials are significant barriers, as the sources of slag and alkali activators are not uniformly distributed geographically and can strongly fluctuate in price. Technical restrictions also play a critical role; for instance, the incompatibility of alkali-activated slag binders with many commercial chemical admixtures used in conventional Portland-cement-based systems poses significant challenges for their adoption in existing construction practices. Moreover, the variability in slag quality from different sources can significantly influence the reactivity and the extent of the activation reaction in alkali-activated slag binders. Differences in slag composition and characteristics can lead to inconsistent performance and properties, creating challenges for quality control, particularly in structural applications.

Safety concerns and current standards further complicate the utilization of these binders. The handling and storage of highly alkaline activators require stringent safety protocols to prevent chemical burns and environmental hazards. Moreover, the prescriptive nature of current standards is a major hindrance. Existing construction codes and standards are primarily designed around traditional Portland-cement-based materials, leaving little room for the incorporation of alternative binders in structural concretes without substantial modifications.

For the aforementioned reasons, several authors have estimated that alternative cements will not be able to exceed 5% of the projected future demand for cementitious materials [6,13,14].

It therefore seems unlikely that alkali-activated materials will displace Portland cement as the primary binder for reinforced concrete in the construction sector [15]. Nevertheless, it is necessary to note that only about 70% of the huge amount of cement produced every year (approximately 3 Gt) is used to manufacture traditional reinforced concrete structures and prefabricated elements. A great deal of cement (more than 1.2 Gt) is used for mortars, plasters and “special” concretes, such as pervious concrete, expansive concrete and smart concrete [16], which can be easily replaced by low-carbon alternative binders such as alkali-activated materials. However, the use of AAS for applications other than ordinary concrete has been only marginally studied by some authors, particularly in the production of lightweight materials [17,18], ultra-high strength materials [19] or those resistant to high temperatures [20]. This paper aims to explore the potential of using this type of binder for the production of structural plasters and pervious concretes.

The experimental research is divided into two main sections:

- Structural plaster (Section 2):

The objective is to develop a premixed M10 alkali-activated slag-based plaster for the structural retrofitting of existing masonry buildings, targeting a 28-day compressive strength of at least 10 MPa. The focus is on elasto-mechanical strength, properties in the fresh state, shrinkage and applicability to masonry. It is worth noting that no relevant papers were found concerning normal-weight AAS plasters in the scientific literature.

- Pervious Concrete (Section 3):

This section explores alkali-activated slag-based pervious concrete utilizing tunnel muck (TM) as a recycled aggregate, in place of natural sand and gravel [21]. In a scenario in which AAS and recycled aggregates are used to produce pervious concretes [22], we evaluate the relationship between aggregate size and the physical and mechanical properties of no-fines concrete made with recycled materials.

## 2. Alkali-Activated Slag-Based Plaster

### 2.1. Materials

**Figure 1:**
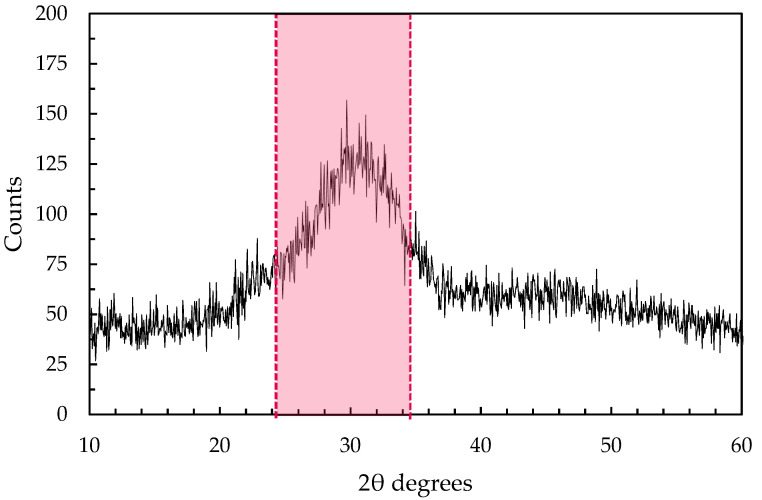
XRD pattern of GGBFS.

Several one-part AAS plasters were prepared by using a ground granulated blast furnace slag (GGBFS) with a specific mass of 3.1 g/cm^3^ and specific surface of 345 m^2^/kg as precursor combined with an alkaline tri-blend solid activator composed of sodium metasilicate pentahydrate, potassium hydroxide and sodium carbonate in a relative mass ratio equal to 7:3:1 in accordance with previous studies by the authors [23,24]. Sodium metasilicate pentahydrate (Silmaco, Lanaken, Belgium), potassium hydroxide (Produits Chimiques de Loos, Aulos, France) and sodium carbonate (Solvay, Rosignano, Italy) were all industrial-grade (total impurities < 1%). The chemical composition of GGBFS is reported in Table 1, while the XRD pattern shows the amorphous nature of the binder as it can be seen from the amorphous hump between 2θ = 25°–35° related to the short-range order of CaO-Al_2_O_3_-MgO-SiO_2_ glass structure (Figure 1).

Four different natural calcareous sands with maximum diameter of 1.5 mm were used as aggregates (with a specific mass in s.s.d. close to 2.5 g/cm^3^ and water absorption lower than 1%), and a calcareous filler was added to the mix to improve the properties of mortars in the fresh state. The water content of plasters was adjusted in order to attain a tixotrophic consistency, identified as a spread on a flow table equal to 170 mm ± 10 mm. No superplasticizers were used to guarantee their proper application to vertical masonry.

In addition, a commercial air-entraining agent was added to the mix for reducing the specific mass of mortars and improving, in combination with a methylcellulose and a modified starch, the fresh-state properties of mixtures (i.e., low level of bleeding and high level of thixotropy). A traditional natural hydraulic lime NHL 3.5 was selected as alternative viscosity modifier to modified starch and methylcellulose, while 6 mm long polypropylene fibres were used to reduce the cracking risk of fresh plasters and an aluminium powder-based expansive agent ensured expansion in plastic phase. Finally, the drying shrinkage of AAS plaster was controlled by means of an ethylene glycol-based shrinkage reducing admixture (SRA) in powder form.

### 2.2. Methods

Several different AAS plasters were prepared in accordance with the “Dry mixing method” reported in [25] and have already been used by the authors in previous studies [26]. They were characterized in the fresh state by measuring their workability by flow table (EN 1015-3 [27]), density (EN 1015-6 [28]), air content (EN 1015-7 [29]) and workable life (EN 1015-9 [30]). Prismatic specimens of 40 mm × 40 mm × 160 mm were cast and kept in laboratory conditions for 24 h (20 ± 2 °C). Then, the specimens were removed from the steel mould, rinsed in water and cured in a climatic chamber (20 ± 2 °C, 60 ± 5% R.H.) till the time of experiment. Compressive strength (EN 1015-11 [31]) was determined 1, 7 and 28 days after casting, and free shrinkage (EN 12617-4 [32]) was measured on prismatic samples stored at 20 ± 2 °C, 60 ± 5% R.H. for 84 days. Finally, application tests were performed by a professional worker on the best-performing plasters applied by trowel on a brick wall to evaluate the applicability of the AAS mortar on vertical surfaces.

### 2.3. First Stage: Effect of Sand-to-Binder Ratio

During the first stage, three different AAS plasters were produced by varying the sand-to-binder ratio between four and six to limit not only the cost of the premixed mortar but also the environmental impact derived from the use of alkaline substances. The activator content was fixed at 8 wt.% vs. the GGBFS mass to ensure a suitable strength of the plaster, while the water dosage was adjusted in order to obtain the same initial workability, equal to 170 mm on the flow table. No admixture was used in this phase as reported in Table 2.

As expected, the workability retention is very pronounced and it is not influenced by the sand-to-binder ratio nor the density in the fresh state, ranging from 2115 to 2100 kg/m^3^. The entrapped air (close to 5%) is only marginally affected by the variation in sand dosage. However, the thixotropy of AAS is very poor and the application of the fresh mortars without viscosity modifiers on the vertical surfaces appears to be very difficult independently of their composition. On the contrary, the elasto-mechanical properties differ greatly among the mortars studied. Twenty-four h after mixing, S8 4:1 reaches a compressive strength close to 5 MPa, while the others are too weak to be removed from the mould. At older ages, the differences between the AAS plasters are still very pronounced, but all the mortars are compliant with the M10 strength class: S8 4:1 has a 28-day compressive strength equal to 24 MPa, S8 5:1 is equal to about 19 MPa and S8 6:1 is close to 17 MPa. Increasing the sand ratio led to slight reductions in the compressive strength of the plasters. Similarly, Chi et al. [33] and Omur et al. [34] investigated the impact of the sand-to-slag ratio on the compressive strength of AASM mixes and discovered that a higher sand-to-slag ratio resulted in a lower compressive strength.

### 2.4. Second Stage: Effect of Viscosity Modifiers

The aim of the second stage was to improve the applicability of AAS plasters by trowel due to the addition of commercial modified starch, methylcellulose and an air-entraining agent, as reported in Table 3. The dosage of the admixtures was fixed for all the mixtures, and the efficiency of the viscosity modifiers was evaluated with applications by trowel on brick panels.

The addition of viscosity modifiers (especially the air-entraining agent) increases the air content of the fresh mixtures and leads to a reduction in the fresh density of the plasters (approximately equal to 10–13% with respect to the mortars without admixtures). At the same time, the viscosity modifiers allow us to increase the workability life while slightly increasing the water dosage to obtain a spreading on the flow table of 170 mm due to the enhanced thixotropy of the mortars (the water content increased from about 13.5 to 15.0 wt.% vs. dry powder mass).

The retarding effect of the viscosity modifiers on the hydration of the slag is evident in the reduced elasto-mechanical properties of the mortars 24 h after casting, which are not suitable for removal from the formwork. After 7 and 28 days, the effect of the admixtures on the strength is also evident and can be ascribed to the higher porosity of the matrix and to a variation in the reaction processes of the slag with the alkaline activators as confirmed by Li et al. [35] and Bai et al. [36]. In general, a reduction of about 35–40% (at 28 days, from 24.0 and 18.9 MPa to 15.4 and 11.3 MPa for mortars with sand-to-binder ratios of 4:1 and 5:1, respectively) can be observed compared to mortars manufactured without viscosity modifiers except for S8 6:1_VM, which shows a decrease in strength close to 70% (from 17.3 to 5.5 MPa).

**Figure 2:**
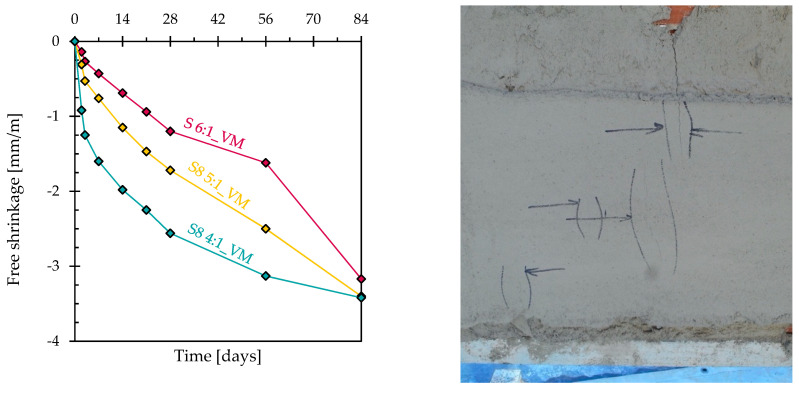
Free shrinkage over time of mortars containing viscosity modifiers (**left**). Cracks in 40 mm thick S8 4:1_VM plaster after a few days from application on brick support (**right**).

The free shrinkage of three plasters (Figure 2, left), even if very different at early ages, is similar after 84 days with values in the range of 3.0–3.5 mm/m, which is totally unacceptable for application in thin sections, such as in plasters, due to the high risk of cracking and detachments, as identified during the application test of 40 mm thin S8 4:1_VM on a vertical brick panel (Figure 2, right).

### 2.5. Third Stage: Effect of Shrinkage-Reducing Admixtures and Environmental Impact

The high shrinkage of AAS plasters requires the addition of admixtures able to reduce the shrinkage of mixtures by half. For this reason, in the third phase, an expansive agent and a shrinkage-reducing admixture were used, alone or in combination, with polymeric fibres able to control the plastic cracking of mortars to produce plasters with a sand-to-binder ratio of 4:1. Moreover, the addition of the expansive agent also improves the applicability of the materials by hand, accelerating the operations for levelling the surface by means of flat floats and straight edges. The composition and the main properties of the three different mortars investigated in this stage are reported in Table 4.

The addition of the aluminium-powder-based expansive agent increases both the workability life and the consistency at 60 min with respect to the S8 4:1_VM plaster while the use of an SRA, alone or in combination with an aluminium-powder-based expansive agent, does not significantly modify the rheological properties of the mixtures. On the contrary, the mortars containing the shrinkage-reducing admixture are characterized, at equal air-entraining agent dosages, by a higher air content and lower density in the fresh state. This determines a strong reduction in compressive strength at younger and older ages, ranging from 50 and 55% vs. plaster without an SRA after 7 and 28 days, respectively. Samples manufactured with an aluminium-powder-based expansive agent (S8 4:1_VM_S and S8 4:1_VM_SE), although this admixture negatively affects their strength, can be classified as M10 mortar.

**Figure 3:**
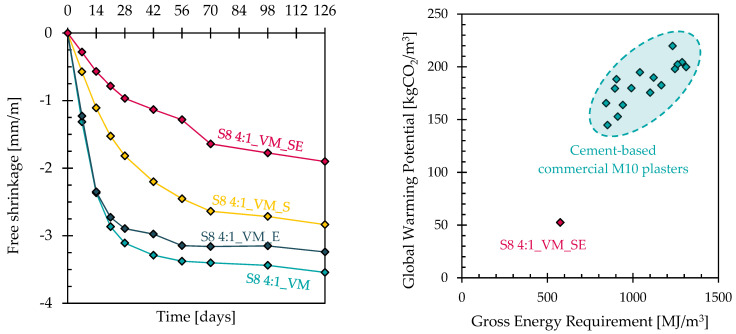
Free shrinkage over time of mortars containing viscosity modifiers and shrinkage-reducing admixtures (**left**). Environmental parameters of S8 4:1_VM_SE and other commercial plasters containing cement at equivalent strength class M10 (**right**). The composition of commercial plasters was provided by the suppliers (**right**).

The effectiveness of the admixtures on the shrinkage reduction is shown in Figure 3 (left). It can be noted that the addition of the aluminium-powder-based expansive agent is responsible for the negligible reduction in the free shrinkage of the plaster over time, while the SRA is capable of limiting the shrinkage marginally. On the other hand, the combined use of the expansive agent and shrinkage-reducing admixture allows us to reduce the shrinkage of the mortar by about 50%, from 3.5 mm/m to 1.8 mm/m after 126 days at 20 °C and 60% R.H., ensuring a crack-free plaster that strongly adheres to the masonry. This behaviour is in full accordance with previous research by the authors [26], Bilek et al. [37] and Al Mokhadmeh and Soliman [38].

The evaluation of the environmental advantages deriving from the use of an AAS plaster instead of traditional Portland-cement-containing premixed mortars was performed by estimating two environmental parameters related to CO_2_ emissions (GWP, global warming potential) and energy requirements (GER, gross energy requirement) using the Ecoinvent 3.0 database. These data, reported in Figure 3 (right) and estimated by taking into account the production process of one cubic meter of premixed mortar, are compared with commercial M10 plasters widely available on the European market and show the reduced environmental impact of the AAS plaster with respect to that of cement-based mixtures. The results, in accordance with the life cycle analyses conducted on AAS materials by different authors [39,40], indicated that the CO_2_ emissions (GWP) are reduced from about 140–230 to about 50 kg_CO2_/m^3^, while the energy consumption (GER) drops from 750–1400 to 600 MJ/m^3^.

## 3. Totally Recycled Pervious Concrete

### 3.1. Materials and Methods

To investigate the influence of aggregate size on the properties of AAS pervious concrete, six different single-sized recycled aggregates from tunnelling works (using drilling and blasting techniques) were used [41]. The properties of the tunnel muck are reported in Table 5. 

An alkali-activated slag-based binder was used to fully replace the Portland cement (the main properties are reported in Section 2.1), while a small amount of an air-entraining agent and 80 kg/m^3^ of natural sand with a maximum size of 0.25 mm were added to the concrete to enhance the porosity and the rheology of the mixtures, respectively. Finally, a commercial PCE superplasticizer was used to obtain a proper consistency in the fresh state. The mix proportions of the pervious concrete are reported in Table 6.

The concrete was prepared in a 350 L concrete mixer, and cubic (with a size of 150 mm) and cylindric (with a diameter of 100 mm and height of 150 mm) specimens were cast in steel moulds with a compacting plate by rodding them 30 times. After 24 h, the samples were placed in a water tank at 20 °C until the tests were conducted. The density of the pervious concrete was measured in accordance with EN 12390-7 [42]; the compressive strength was determined on a set of three cubic samples after 1, 7 and 28 days with a constant loading rate of 0.6 MPa/s according to EN 12390-3 [43]; and the water permeability (six samples for each methodology) was tested after 28 days by using the constant head (CH) and falling head (FH) methods (ACI 522R-10 [44]). During the constant head test, the maximum steady water flow through the specimen was measured by weighing the water volume flowing through the pervious concrete at a fixed time interval. The permeability coefficient K_CH_ is estimated by means of Darcy’s law with the following equation:

(1) $$K_{CH}=\frac{Q}{∆t}\cdot \frac{H}{S\cdot h_{f}}$$

where:

*Q/*Δ*t* is the steady water flow measured during the test;

*H* is the height of the concrete sample equal to 150 mm;

*S* is the section of the concrete samples equal to 7854 mm^2^ (a 100 mm diameter);

*h_f_* is the head of water above the sample equal to 300 mm.

On the contrary, the falling head method is based on the measurement of the time required to drop a column of water with a height of 290 mm through the sample. In this case, the permeability coefficient K_FH_ is estimated with the following equation in accordance with ACI 522R-10 [44]:

(2) $$K_{FH}=\frac{S_{1}l}{S_{2}t}\cdot log\frac{h_{2}}{h_{1}}$$

where:

*S*_1_ is the section of the test apparatus equal to 7854 mm^2^ (a 100 mm diameter);

*S*_2_ is the section of the concrete sample equal to *S*_1_;

*l* is the height of the concrete samples equal to 150 mm;

*h*_1_ and *h*_2_ are the initial and final head of water above the sample, equal to 300 and 10 mm, respectively;

*t* is the measured time to reach the final head of water *h*_2_.

#### Experimental Results

**Figure 4:**
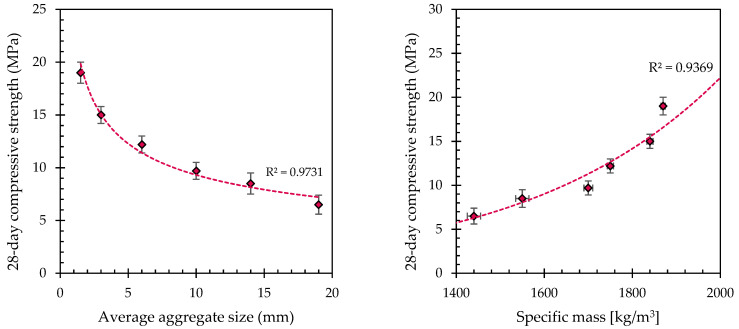
Compressive strength over 28 days as a function of the aggregate size (**left**). Correlation between specific mass and compressive strength of different pervious concretes 28 days after casting (**right**).

Table 7 and Figure 4 (left) show the test results for the compressive strength of the samples. After 28 days, the pervious concretes containing small-sized aggregates achieve compressive strengths ranging from 12 to 19 MPa, while the concretes manufactured with coarse aggregates with a size greater than 8 mm evidence compressive strengths lower than 10 MPa. It has been observed that increasing the aggregate size leads to a reduction in compressive strength at both younger and older ages, consistent with the findings reported by Fu et al. [45], Chockalingam et al. [46] and Yahia and Kabagire [47] for no-fines concretes with natural gravel. In conventional concrete, the interfacial transition zone (ITZ) is the most critical factor influencing strength, as it is typically the weakest part of the concrete. Conversely, in pervious concrete, the designed voids represent the weakest component. As the aggregate size increases, the volume of these voids also increases beyond the expected levels, resulting in a decrease in compressive strength. The results of specific mass measurements corroborate this observation, showing that the density is strongly influenced by the average diameter of recycled aggregates: PC 1-2 and PC 2-4 are close to 1850 kg/m^3^; PC 4-8 and PC 8-12 are in the range of 1700–1750 kg/m^3^; and PC 12-16 and PC 16-22 show a lower specific mass, equal to 1550 and 1440 kg/m^3^, respectively. This determines a close correlation between density and resistance, as shown in Figure 4 (right).

**Figure 5:**
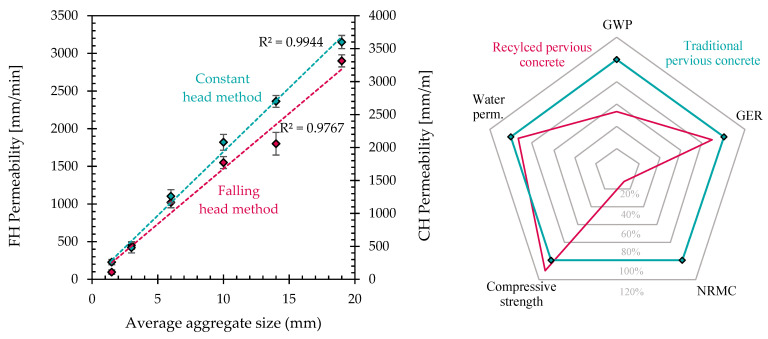
Water permeability coefficient of different pervious concretes as a function of aggregate size (**left**). Environmental parameters (GWP, GER and NRMC) and main performance (water permeability and compressive strength) of recycled pervious concrete normalized with respect to a traditional Portland-cement-based pervious concrete (**right**).

Analysing the water permeability results (Table 7), several conclusions can be drawn. First of all, both the FH and CH methods showed the same permeability trend in the tested pervious concretes, even if the value of K_FH_ differs from K_CH_ due to different pressures during the test. Mixtures containing large aggregates show a more pronounced permeability that is, in general, one order of magnitude higher with respect to those of PC 1-2 and PC 2-4, which are manufactured with small-sized recycled gravel. In particular, with the FH methods, it is possible to estimate a permeability coefficient ranging from 100–500 mm/min to 2000–3000 mm/min, while the coefficient K_CH_ varies from 250–500 mm/min to 2500–3500 mm/min. Similarly to the other physical properties of recycled pervious concrete, the water permeability is also strictly related to the aggregate size: the bigger the aggregate, the higher the permeability coefficients (Figure 5, left).

Finally, the estimation of the environmental parameters of recycled AAS pervious concrete at a similar compressive strength class and with a similar level of water permeability shows the considerable advantages of the replacement of Portland cement with alkali-activated slag and natural aggregates with tunnel muck (Figure 5, right). In detail, the CO_2_ emissions (GWP) decrease by about 50%, and the natural raw material consumption (NRMC) is reduced by more than 80%, while limited advantages can be highlighted in terms of the energy consumption to produce one cubic meter of pervious concrete.

## 4. Conclusions

This study explores the feasibility of using alkali-activated slag (AAS) as a sustainable binder for pervious concrete and structural plaster. The findings demonstrate the potential of AAS in developing eco-friendly construction materials that can replace traditional Portland cement-based products, thereby reducing the environmental impact of the construction industry. Key conclusions drawn from the research are as follows:

- Structural Plaster
  ○ The developed AAS-based plaster achieved a 28-day compressive strength of at least 10 MPa, making it suitable for the structural retrofitting of masonry buildings;
  ○ Its fresh-state properties, shrinkage and applicability to masonry were thoroughly investigated, ensuring the practicality of the plaster in real-world applications;
  ○ The addition of viscosity modifiers, including methylcellulose and modified starch, improved its applicability by trowel but resulted in a reduction in compressive strength (on average, by −40% with respect to the non-admixed mortars) due to the increased air content and altered hydration processes;
  ○ The use of shrinkage-reducing admixtures successfully mitigated the high level of shrinkage associated with AAS plasters, making them viable for thin-section applications.
- Pervious Concrete
  ○ AAS-based pervious concrete was successfully manufactured using tunnel muck (TM) as a recycled aggregate, demonstrating a sustainable approach to aggregate utilization;
  ○ The relationship between aggregate size and the physical and mechanical properties of no-fines concrete was evaluated, confirming the correlations evidenced for traditional cement-based pervious concretes;
  ○ The developed pervious concrete exhibited a compressive strength up to about 20 MPa and permeability ranging from 500 to 3000 mm/min, making it suitable for applications requiring enhanced drainage and reduced surface runoff.

Overall, the research underscores the promise of alkali-activated slag as a versatile and sustainable binder for both pervious concrete and structural plaster applications with an environmental impact, at equal levels of performance, reduced by about 50 to 75% and 10 to 30% in terms of GWP and GER, respectively. Further studies are encouraged to optimize the formulations and address any remaining challenges to facilitate the broader adoption of AAS-based materials in construction practices.

## Figures and Tables

**Table 1:** Chemical composition of GGBFS.

	Component wt.%								
	CaO	Al_2_O_3_	SiO_2_	Fe_2_O_3_	SO_3_	TiO_2_	K_2_O	MgO	Others
GGBFS	45.8	10.0	32.8	1.5	0.2	2.0	0.5	6.4	0.8

**Table 2:** Composition and main properties of AAS plasters.

	S8 4:1	S8 5:1	S8 6:1
GGBFS (kg/m^3^)	365	305	260
Calcareous filler (kg/m^3^)	145	150	155
Sand (kg/m^3^)	1320	1375	1410
Alkaline activators (kg/m^3^)	29	24	21
Water (kg/m^3^)	256	253	252
Initial workability (mm)	170	170	170
Workability at 60 min (mm)	150	160	160
Workability life (min)	110	110	120
Air content (%)	4.5	5.0	5.0
Density of fresh mortar (kg/m^3^)	2115	2105	2100
1-day compressive strength (MPa)	4.9	-	-
7-day compressive strength (MPa)	17.6	15.3	14.0
28-day compressive strength (MPa)	24.0	18.9	17.3

**Table 3:** Composition and main properties of AAS plasters containing viscosity modifiers.

	S8 4:1_VM	S8 5:1_VM	S8 6:1_VM
GGBFS (kg/m^3^)	315	245	205
Calcareous filler (kg/m^3^)	125	120	120
Sand (kg/m^3^)	1135	1105	1110
Alkaline activators (kg/m^3^)	25	20	16
Water (kg/m^3^)	240	220	215
Methylcellulose	0.03 wt.% vs. dry powder mass		
Modified starch	0.01 wt.% vs. dry powder mass		
Air entraining agent	0.04 wt.% vs. dry powder mass		
Initial workability (mm)	170	170	170
Workability at 60 min (mm)	165	165	170
Workability life (min)	>120	>120	>120
Air content (%)	15	19	20
Density of fresh mortar (kg/m^3^)	1850	1710	1680
1-day compressive strength (MPa)	-	-	-
7-day compressive strength (MPa)	13.1	9.2	5.1
28-day compressive strength (MPa)	15.4	11.3	5.5

**Table 4:** Composition and main properties of AAS plasters containing viscosity modifiers and admixtures for shrinkage reduction.

	S8 4:1_VM	S8 4:1_VM_S	S8 4:1_VM_E	S8 4:1_VM_SE
GGBFS (kg/m^3^)	315	290	325	300
Calcareous filler (kg/m^3^)	125	115	130	120
Sand (kg/m^3^)	1135	1045	1170	1080
Alkaline activators (kg/m^3^)	25	23	26	24
Water (kg/m^3^)	240	210	235	220
Methylcellulose	0.03 wt.% vs. dry powder mass			
Modified starch	0.01 wt.% vs. dry powder mass			
Air entraining agent	0.04 wt.% vs. dry powder mass			
Polymeric fibres (wt.% vs. dry powder mass)	-	0.06	0.06	0.06
Expansive agent (wt.% vs. dry powder mass)	-	-	0.08	0.08
Shrinkage reducing admixture (wt.% vs. binder mass)	-	0.50	-	0.50
Initial workability (mm)	170	170	170	170
Workability at 60 min (mm)	165	145	170	150
Workability life (min)	>120	120	>>120	>120
Air content (%)	15	20	13	18
Density of fresh mortar (kg/m^3^)	1850	1680	1900	1755
1-day compressive strength (MPa)	-	-	-	-
7-day compressive strength (MPa)	13.1	6.5	11.5	8.9
28-day compressive strength (MPa)	15.4	7.0	12.3	10.8

**Table 5:** Main properties of recycled aggregates.

	TM 1-2	TM 2-4	TM 4-8	TM 8-12	TM 12-16	TM 16-22
Size (mm)	1–2	2–4	4–8	8–12	12–16	16–22
Specific mass s.s.d. (g/cm^3^)	2.65	2.65	2.64	2.65	2.64	2.64
Water absorption s.s.d. (%)	2.0	1.9	2.0	1.7	1.5	1.2
Flakiness index	-	-	FI_15_	FI_15_	FI_15_	FI_15_
Shape index	-	-	SI_15_	SI_15_	SI_15_	SI_15_

**Table 6:** Composition of pervious concretes.

	PC 1-2	PC 2-4	PC 4-8	PC 8-12	PC 12-16	PC 16-22
GGBFS (kg/m^3^)	350	350	350	350	350	350
Alkaline activators (kg/m^3^)	70	70	70	70	70	70
TM 1-2 (kg/m^3^)	1450	-	-	-	-	-
TM 2-4 (kg/m^3^)	-	1450	-	-	-	-
TM 4-8 (kg/m^3^)	-	-	1450	-	-	-
TM 8-12 (kg/m^3^)	-	-	-	1450	-	-
TM 12-16 (kg/m^3^)	-	-	-	-	1450	-
TM 16-22 (kg/m^3^)	-	-	-	-	-	1450
Natural sand (kg/m^3^)	80	80	80	80	80	80
Water (kg/m^3^)	110	110	110	110	110	110
Air entraining agent (g/m^3^)	20	20	20	20	20	20
Superplasticizer (kg/m^3^)	5.0	4.2	3.5	1.75	-	-

**Table 7:** Main properties of pervious concretes (average values).

	PC 1-2	PC 2-4	PC 4-8	PC 8-12	PC 12-16	PC 16-22
1-day compressive strength (MPa)	10.7	9.5	6.0	7.2	6.5	5.0
7-day compressive strength (MPa)	12.0	11.5	7.5	8.0	7.0	6.0
28-day compressive strength (MPa)	19.0	15.0	12.2	9.7	8.5	6.5
Specific mass (kg/m^3^)	1870	1840	1750	1700	1550	1440
Permeability K_FH_ (mm/min)	95	450	1020	1550	1800	2900
Permeability K_CH_ (mm/min)	260	480	1260	2080	2700	3600

## Data Availability

The original contributions presented in the study are included in the article, further inquiries can be directed to the corresponding author.
